# Partially Hydrolysed Whey-Based Infant Formula Improves Skin Barrier Function

**DOI:** 10.3390/nu13093113

**Published:** 2021-09-04

**Authors:** Sébastien Holvoet, Sophie Nutten, Lénaïck Dupuis, Dominique Donnicola, Tristan Bourdeau, Betsy Hughes-Formella, Dagmar Simon, Hans-Uwe Simon, Ryan S. Carvalho, Jonathan M. Spergel, Sibylle Koletzko, Carine Blanchard

**Affiliations:** 1 Department of Gastrointestinal Health, Nestlé Institute of Health Sciences, Nestlé Research, Société des Produits Nestlé S.A., Vers-chez-les-Blanc, 1000 Lausanne, Switzerland; Sebastien.holvoet@rdls.nestle.com (S.H.); Sophie.nutten@nestle.com (S.N.); dominique.donnicola@rdls.nestle.com (D.D.); Tristan.Bourdeau@rdls.nestle.com (T.B.); 2 Biostatistics and Data Management, Clinical Research Unit, Nestlé Research, Société des Produits Nestlé S.A., Vers-chez-les-Blanc, 1000 Lausanne, Switzerland; Lenaick.Dupuis@rdls.nestle.com; 3 External Consultant, 21465 Reinbek, Germany; betsyhughesformella@me.com; 4 Department of Dermatology, Inselspital, Bern University Hospital, 3010 Bern, Switzerland; Dagmar.Simon@insel.ch; 5 Institute of Pharmacology, University of Bern, 3012 Bern, Switzerland; hans-uwe.simon@pki.unibe.ch; 6 Department of Clinical Immunology and Allergology, Sechenov University, 119991 Moscow, Russia; 7 Laboratory of Molecular Immunology, Institute of Fundamental Medicine and Biology, Kazan Federal University, 420008 Kazan, Russia; 8 Institute of Biochemistry, Medical School Brandenburg, 16816 Neuruppin, Germany; 9 Nestlé Nutrition, 1800 Vevey, Switzerland; Ryan.Carvalho@nestle.com; 10 Department of Pediatrics, Children’s Hospital of Philadelphia, Perelman School of Medicine at University of Pennsylvania, Philadelphia, PA 19104, USA; SPERGEL@email.chop.edu; 11 Department of Pediatrics, Dr. von Hauner Children’s Hospital and University Hospital, LMU Munich, 80337 Munich, Germany; Sibylle.Koletzko@med.uni-muenchen.de; 12 Department of Pediatrics, Gastroenterology and Nutrition, School of Medicine Collegium Medicum, University of Warmia and Mazury, 10-719 Olsztyn, Poland

**Keywords:** aquaporin, partially hydrolysed whey-based infant formula, skin barrier function, transepidermal water loss

## Abstract

Specific partially hydrolysed whey-based infant formulas (pHF-W) have been shown to decrease the risk of atopic dermatitis (AD) in infants. Historically, AD has been associated primarily with milk allergy; however, defective skin barrier function can be a primary cause of AD. We aimed to ascertain whether oral supplementation with pHF-W can improve skin barrier function. The effect of pHF-W was assessed on transepidermal water loss (TEWL) and antibody productions in mice epicutaneously exposed to *Aspergillus fumigatus*. Human primary keratinocytes were stimulated in vitro, and the expression of genes related to skin barrier function was measured. Supplementation with pHF-W in neonatal mice led to a significant decrease in TEWL and total IgE, but not in allergen-specific antibody levels. The whey hydrolysate was sufficient to decrease both TEWL and total IgE. Aquaporin-3 gene expression, linked with skin hydration, was modulated in the skin of mice and human primary keratinocytes following protein hydrolysate exposure. Skin barrier improvement may be an additional mechanism by which pHF-W may potentially reduce the risk of AD development in infants. Further human studies are warranted to confirm the clinical efficacy of these observations.

## 1. Introduction

Atopic dermatitis (AD) is the most prevalent allergic disease in the first year of life, affecting 5 to 20% of infants and toddlers [1] and is associated with morbidities later in childhood. Clinical trials in infants at risk of developing AD have shown that, when exclusive breastfeeding is not possible, supplementation with specific partially hydrolysed whey-based infant formulas (pHF-W) significantly reduces infant risk of developing AD as compared to intact protein formula [2]. However, the mechanism by which specific pHF-W reduces the risk of AD development [3,4] is not well described and may be informed by the etiology of AD disease.

Defective skin barrier function is a well-documented risk factor for skin diseases, including AD. Recent genome-wide association studies suggest that specific mutations in genes encoding for skin barrier function molecules are strongly linked with AD risk [5]. Notably, loss-of-function variants in the filaggrin gene, a filament binding protein of the stratum corneum, have been identified as a key risk factor for AD [5,6,7,8,9,10,11,12,13]. Additional studies have demonstrated that increased transepidermal water loss (TEWL) in the first weeks of life was associated with an increased risk of AD development, which was independent of family history [14], yet the retraction of a key publication added controversy to the field [15]. Furthermore, recent evidence in murine studies has demonstrated that allergen application (including ovalbumin, peanut, milk protein, or *Aspergillus fumigatus*) on an impaired barrier can lead to systemic sensitisation [16,17,18,19,20]. These studies, along with genetic studies linking filaggrin mutations with food allergy [21], have led to the hypothesis that allergic sensitisation may be secondary to AD or skin barrier impairment. AD is thus now recognised as a major risk factor for the development of food allergy [22], and as a consequence, preventive strategies for food allergy may require a primary prevention of AD or skin barrier impairment [23].

AD prevention has been explored with various nutritional and topical approaches. The efficacy of pHF-W supplementation on AD risk reduction has been tested in clinical settings with numerous product types, and the meta-analyses of these studies produced conflicting results [24,25,26,27,28]. However, two recent meta-analyses with a specific pHF-W demonstrated its efficacy at reducing the risk of AD development [2,29]. Yet, the mechanism by which pHF-W may reduce AD risk is highly debated. In a context where AD is a skin disease before being a food allergy disease, unappreciated mechanisms, beyond a decreased exposure to milk allergen and oral tolerance induction [3,18], may play a role in the clinical reduction in AD risk observed with specific pHF-W as compared to intact protein infant formula (IF). Overall, the mounting knowledge involving the skin barrier hypothesis in AD etiology, and the recent demonstration that pHF-W may be effective in reducing the risk of AD in the general infant population with a similar effect size as at-risk for allergy infants [29], led to the hypothesis that pHF-W efficacy may be linked to a milk allergen-independent mechanism such as improvement in skin barrier function.

Here, we describe a pre-clinical model that was employed to test the efficacy of a specific pHF-W on skin barrier function and allergen-specific immunoglobulin levels. We used a non-milk-related allergen (*Aspergillus fumigatus, Af)* to dissociate the benefit that pHF-W may have on milk-specific oral tolerance induction from potentially beneficial effects on skin barrier function. We demonstrated that in addition to inducing oral tolerance [4], pHF-W can also improve skin barrier function, thereby protecting the skin from non-milk-related entries of allergens. Our findings may explain one of the possible mechanisms by which pHF-W may prevent AD development in infants.

## 2. Materials and Methods

### 2.1. Murine Model for Skin Barrier Impairment in Neonatal Mice

**Figure 1:**
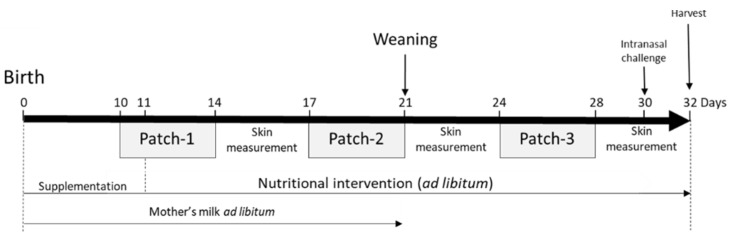
Atopic dermatitis model in neonatal mice and nutritional intervention: (**a**) neonatal mice were exposed to *Af* protein extract epicutaneously with 3 consecutive patches. Mice were challenged intranasally with *Af* day 30. The nutritional intervention was performed over the experiments with either intact protein formula (IF), partially hydrolysed formula (pHF-W), hydrolysate, or the lipid blend. Mice receiving nutritional interventions were all exposed to *Af.* The tissues were harvested on day 32.

The animal study protocol VD3059 was approved by the Service Vétérinaire du Canton de Vaud, Switzerland. Briefly, ten-day-old neonatal mice (BALB/cByJ JAX TM strain from Charles River, L’Arbresle, France) were exposed to *Af* protein extract via a skin patch on the back of the animal (Figure 1).

Each patch was applied for 5 consecutive days before removal. In total, 3 patches were applied with 3 days between each patch exposure, beginning 10 days after birth. The skin exposure was performed under isoflurane (Baxter, Volketswil, Switzerland) anaesthesia. The hair on the back skin was clipped closely with an electric clipper when needed and the skin was cleaned with 70% isopropanol solution (VWR, Nyon, Switzerland). Amounts of 20 µL (patch-1), 50 µL (patch-2) and 100 µL (patch-3) of *Af* protein extract (Greer Laboratories, Lenoir, NC, USA) at 2 mg/mL were applied in the sensitised groups (S.) and NaCl 0.9% (Merck, Zug, Switzerland) was applied in the non-sensitised (N.S.) group on a 0.3 cm^2^ (patch-1), 0.5 cm^2^ (patch-2) and 1 cm^2^ (patch-3) patch of sterile gauze (Hartmann Dermaplast, Chatenois, France) secured on the skin with a bio-occlusive, waterproof transparent dressing (Systagenix Bioclusive, San Antonio, Texas, USA) and a Band-Aid (Mefix, Wasquehal, France). Mice were challenged intranasally under isoflurane anaesthesia on day 30 with 100 µg of *Af* diluted in 0.9% NaCl and euthanatised on day 32. It is important to note that our models do not involve tape stripping or other methods of skin abrasion. The clipping and isopropanol skin preparation have been shown, by us and others, to be enough in adult mice to induce systemic sensitisation with *Af*, house dust mite extract, and beta-lactoglobulin (BLG) ([19,20,30] and data not shown).

### 2.2. Nutritional Intervention

Neonate mice were fed from the first day of life to their 11th day with supplementation of 10 to 100 µL of IF (Beba Optipro1, NWSB003, Nestlé, Switzerland); pHF-W (Beba-HA1, NWHSB 228, Nestlé Switzerland), hydrolysate, or lipid blend (Nestlé Factory, Bissenhofen, Germany), or plain water (control groups N.S. and S.), daily. The hydrolysate and lipid blend were composed of the exact ingredients used in the preparation of the pHF-W infant formula. Ingredients were prepared in water and based on the protein content and lipid content of pHF-W. This was in addition to the mother’s breast milk. On day 11, the pups received the formula ad libitum. At weaning, the pups were separated from their mothers, and formulas were given in a drinking bottle and changed every day. Formulas (IF and pHF-W) were prepared according to the manufacturer’s recommended reconstitution dose (at the concentration of 146 mg/mL). The mothers were fed a milk-free diet.

### 2.3. Transepidermal Water Loss (TEWL)

TEWL was measured in the patch area once a day, 1 to 3 h after patch removal with a DermaLab Combo (Cortex Technology, Hadsund, Denmark) on day 14 to 17, day 21 to 24, and 28 to 32 for the neonate mice model (Figure 1). TEWL is used to characterise skin barrier function. It is the amount of water that passively evaporates through the skin due to the water vapor pressure gradient on both sides of the skin barrier [31].

### 2.4. Broncho-Alveolar Lavage (BAL)

BAL fluids were collected in 1 mL of phosphate-buffered saline solution (PBS) (Merck & Cie, Schaffaussen, Switzerland) containing 0.2% Bovine Serum Albumin (BSA) (Merck & Cie Schaffaussen, Switzerland). BAL fluids were centrifuged for 10 min at 2000 rpm at 4 °C. Cell pellets were suspended in 150 µL of PBS and 0.2% BSA and transferred on slides by cytospin for 5 min at 500 g. Cells were stained using the DIFF Quick staining kit (Medion Diagnostics, Miami, FL, USA) following the manufacturer’s instructions to differentiate cell types and quantified.

### 2.5. Total and Af-Specific IgE and IgG1

Total IgE levels were quantified by ELISA (BD Biosciences, Allschwil, Switzerland) following the manufacturer’s protocol. Specific IgE and IgG1 levels were quantified as previously described [30].

### 2.6. Human Keratinocytes Cell Culture

A pool of 4 x 10^4^ primary human epidermal keratinocytes (CELLnTec, Bern, Switzerland) were seeded in a 48-well plate (Corning, VWR, Nyon, Switzerland) in CnT57 medium (CELLnTec, Bern, Switzerland) supplemented with 10% serum. When keratinocytes reached 90% confluence, cells were treated with the hydrolysate at 1 µg protein/mL. One hour after the addition of the ingredient, 50 ng/mL of interleukin-13 (IL-13) (Peprotech, London, UK) was added and incubated at 37 °C with 5% CO_2_ for 48 h. An identical volume of medium was added to the non-IL-13 primed cultures.

### 2.7. Quantitative Gene Expression Levels by Real-Time PCR

Total RNA was extracted from mouse skin (epidermis, dermis, adipose and muscle layers) and in vitro stimulated keratinocytes with the RNeasy mini kit (Qiagen, Hombrechtikon Switzerland) according to the manufacturer’s instructions. Reverse transcription was performed on 0.5 µg of total RNA by using the Qscript CDNA supermix kit (Quantabio, VWR, Nyon Switzerland) according to the manufacturer’s protocol. Taqman probes from Applied Biosystem (Basel, Switzerland) were used to quantify Mouse *aquaporin-3* (Mm01208559_m1), Human Filaggrin (Hs00856927_g1), Human Aquaporin-11 (*AQP11*: Hs00542681_m1), Human Involucrin (Hs00846307_s1), Human Calpain-14 (Hs00871882_m1), Human Desmoglein-1 (Hs00355084_m1) and Human Eotaxin-3 (*CCL-26*; Hs00171146_m1). Quantification was normalised with the mean of 2 housekeeping genes (β-actin (Hs01060665_m1) and Glyceraldehyde 3-phosphate dehydrogenase (*GAPDH*) (Hs02758991_m1)) for human keratinocytes, and 3 housekeeping genes (hypoxanthine phosphoribosyl-transferase (*Hprt*, Mm01545399_m1), *Gapdh* (Mm99999915_g1) and β-actin (Mm00607939_s1) (Applied Biosystem)) for mouse skin experiments. Real-time PCR was performed on ABI PRISM 7900HT (Applied Biosystem). Calculations were performed on the cycle threshold (Ct) values. The Ct value for each gene was corrected by the Ct mean of the two or three housekeeping genes. The results were calculated as a relative expression and expressed as arbitrary units using the formula 2^−ΔCt^ × K where K is a 10^3^ factor.

### 2.8. Statistics

Preclinical parameters were described by the median (interquartile range) or median + standard error (Rouseeuw robust estimator) and were analysed using non-parametric statistics. The exact Wilcoxon rank-sum test was applied to determine whether the specified comparisons were statistically significantly different. One-sided *p*-values were provided when Af-exposed (S.) mice were compared to non-exposed mice (i.e., sensitisation model: S. > N.S.). Otherwise, two-sided *p*-values were provided. Probability values of less than 5% were considered statistically significant (*p* < 0.05). The analyses were performed using GraphPad Prism (GraphPad Software, San Diego, CA, USA) and R 3.4.1 or higher (R core Team, Vienna, Austria). Values from in vitro data are expressed as mean ± SD and statistical analyses were performed using Welch’s t-test.

## 3. Results

### 3.1. pHF-W Reduces TEWL and Total IgE Levels Following Af Skin Exposure in Neonatal Mice

**Figure 2:**
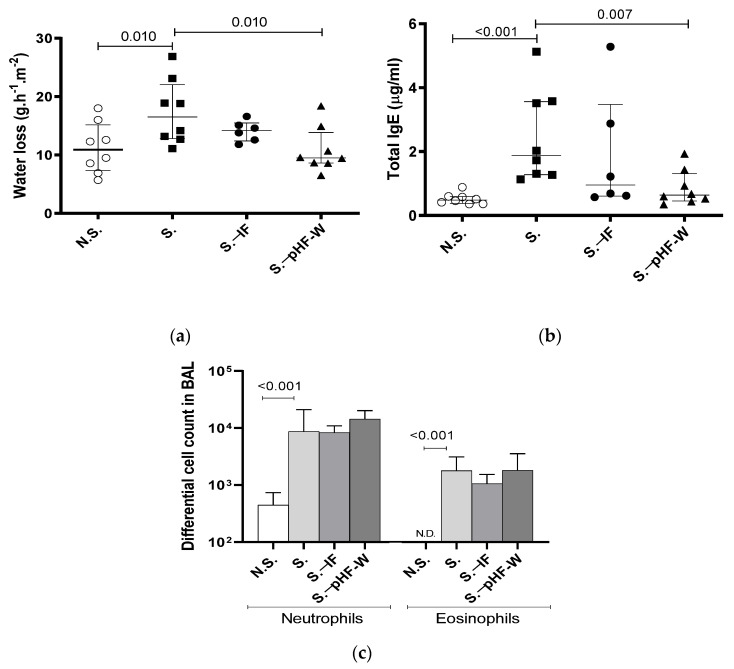
Effect of pHF-W and IF on TEWL and total IgE levels and lung inflammation in neonatal mice following exposure to *Af:* (**a**) TEWL, (**b**) total IgE, and (**c**) the numbers of neutrophils and eosinophils in the bronchoalveolar lavage (BAL) following an *Af* challenge are shown. Data are expressed as median ± (interquartile range). *N* = 8 for the N.S. group of mice non-exposed to *Af* (open circle) and S. group exposed to *Af* (black square), *n* = 6 for S.-IF group exposed to *Af* and supplemented with IF (black circle) and n = 8 for S. −pHF-W group exposed to *Af* and supplemented with pHF-W (black triangle). N.S., Non-sensitised, S., Sensitised, S.−IF, Sensitised supplemented with intact protein infant formula, S. −PHF-W, sensitised supplemented with partially hydrolyzed formula.

To test whether pHF-W could modulate skin barrier function, we examined the effect of pHF-W in the Af-exposure model. TEWL showed significant differences in the *Af*-exposed (S.) group as compared to the N.S. group receiving a patch with saline (17 (13–22) vs. 11 (7–15) g/m^2^/h; *p* = 0.010) after the third patch (Figure 2a).

In this context, pHF-W significantly reduced TEWL compared to the non-supplemented sensitised (S.) mice (17 (13–22) vs. 10 (9–14) g/m^2^/h; *p* = 0.010) (Figure 2a). Importantly, over the entire time course of the experiments, the neonatal mice did not develop any visible skin symptoms across all groups. Total IgE was significantly increased in the group exposed to *Af* as compared to the N.S. group (1.876 (1.278–3.562) vs. 0.483 (0.375–0.592) µg/mL; *p* < 0.001) (Figure 2b). Oral pHF-W administration significantly reduced the total IgE levels (0.636 (0.454–1.307) vs. 1.876 (1.278–3.562) µg/mL; *p* = 0.007) when compared to the non-supplemented S. group (Figure 2b). The anti-*Af*-specific IgE were mostly below quantifiable levels in this model. The *Af*-specific IgG1 levels were increased in the model, supporting some level of specific sensitisation, but were not significantly decreased by the formulas (data not shown). None of the formulas were not able to decrease the *Af*-induced allergic airway cell infiltrates in this model (Figure 2c) based on eosinophil numbers in the lungs. Interestingly, a slight decrease in TEWL and total IgE was observed with IF, but the effect was consistently not significant in the different experiments and the effect-size was always smaller than the one of pHF-W, suggesting that pHF-W significantly decreases TEWL and the total IgE, while IF does not. Taken together, these data suggest that, in this model, oral pHF-W administration has a significant effect on skin barrier function.

### 3.2. TEWL Reduction Occurs following Oral Hydrolysate Supplementation in Neonatal Mice

**Figure 3:**
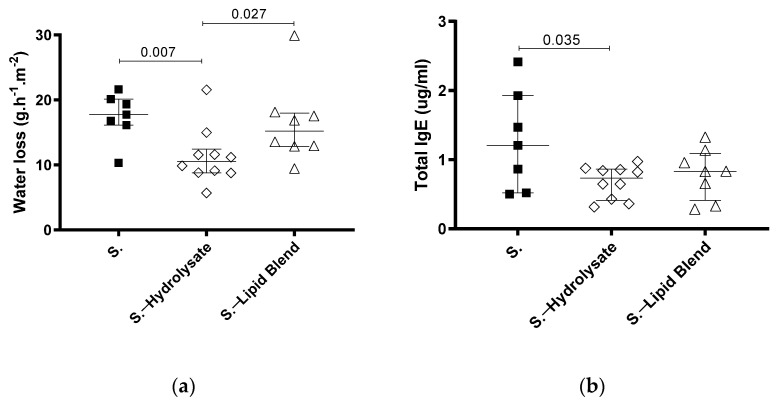
Effect of supplementation with hydrolysate and lipid blend on TEWL in neonatal mice following *Af-*exposure. (**a**) TEWL was assessed at day 30 and (**b**) total IgE was quantified. Data are expressed as median ± interquartile ranges. *Af*-exposed animals (S.) received hydrolysate (empty diamond) or the lipid blend (empty triangle), from birth to day 32. *N* = 7 for S., *n* = 10 for S.—hydrolysate, and *n* = 8 for S.—lipid blend.

Since we were able to identify that pHF-W could decrease TEWL, we investigated which components in the formula were sufficient to provide this benefit. The lipid blend and the hydrolysed proteins (hydrolysate) are the two main differences between IF and pHF-W that could explain this observation. Therefore, we tested whether supplementation with only the hydrolysate or the lipid blend could influence TEWL in the neonatal mice. The mice supplemented with the hydrolysate had significantly lower TEWL than the S. group (11 (9–12) vs. 18 (16–20) g/m^2^/h; *p* = 0.007) and the oral lipid blend exposed group (11 (9–12) vs. 15. (13–18) g/m^2^/h; *p* = 0.027) (Figure 3a).

The hydrolysate supplementation (S.—hydrolysate) also led to significantly lower total IgE than in the S. group (1.2 (0.5–1.9) vs. 0.7 (0.4–0.9) ug/mL; *p* = 0.035) (Figure 3b). Even though the lipid blend was sufficient to decrease the total IgE, the effect was not significant. These results imply that the partially hydrolysed whey hydrolysate is an active component in pHF-W that beneficially modulates the skin barrier function in neonatal mice.

### 3.3. The Expression of Aquaporin-3 Gene Is Modulated by Oral Hydrolysate Supplementation

**Figure 4:**
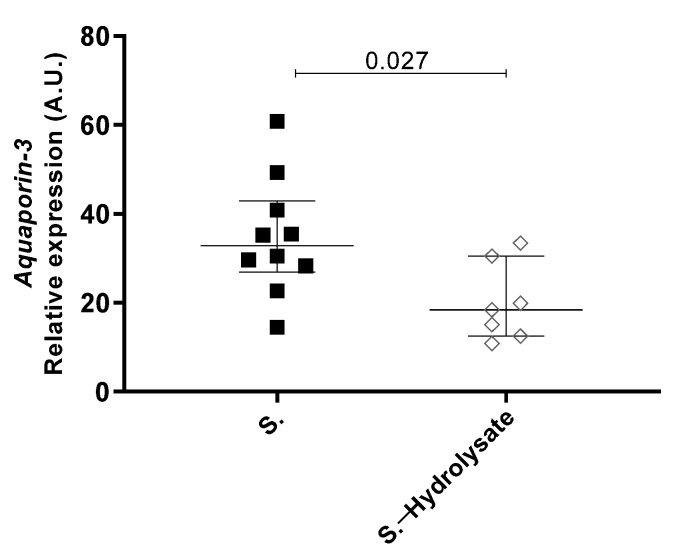
Effect of supplementation with the hydrolysate on aquaporin-3 gene expression in the skin. Murine aquaporin-3 mRNA expression level was measured at the site of *Af*-exposure. Data are expressed as median ± interquartile range with n= 8 for non-supplemented mice (S.) and n = 7 for the mice supplemented the hydrolysate (S.—hydrolysate).

The increased expression of murine *aquaporin 3* (*Aqp3*) has been previously associated with increased TEWL and skin hydration [32]. In our neonatal mouse model, *Aqp3* was significantly decreased in the group supplemented with hydrolysate compared to the non-supplemented *Af-*exposed mice (Figure 4). Taken together, these results suggest that the pHF-W and the hydrolysate may contribute to the skin barrier function and water transport in vivo by specifically and differentially regulating the aquaporin-3 gene.

### 3.4. The Expression of Aquaporin Genes Is Modulated by the Hydrolysate In Vitro

**Figure 5:**
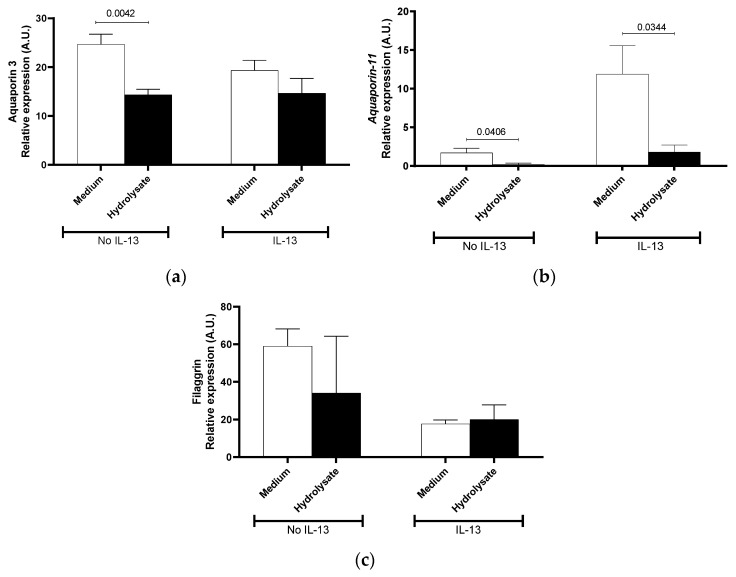
Effect of supplementation with IF, pHF-W, and the hydrolysate on skin barrier gene expression in vitro in primary human keratinocytes. Human (**a**) aquaporin-3, (**b**) aquaporin-11 (**c**) filaggrin mRNA levels were measured in primary human keratinocytes stimulated or not with IL-13. Bar charts represent the mean of 3 triplicates with SD. In vitro effects were repeated in 3 separate experiments.

The direct effect of the hydrolysate on primary human skin keratinocytes was then investigated. *AQP3* and *AQP11*, filaggrin *(FLG),* involucrin, desmoglein, calpain-14, small proline-rich protein *(SPRR),* thymic stromal lymphopoietin *(TSLP),* and C-C Motif Chemokine Ligand 26 *(CCL26)* mRNA expression were measured in human primary skin keratinocytes in the presence or absence of IL-13 stimulation in vitro. We observed that the two members of the *Aquaporin* gene family were differentially decreased when non-stimulated cells were exposed to the hydrolysate (Figure 5a,b). For *AQP3*, this decrease was only observed in non-stimulated keratinocytes following exposure with the hydrolysate (Figure 5a) (*p* = 0.042).

In IL-13-stimulated keratinocytes, the *AQP11* mRNA levels increased (7-fold) and a significant decrease following exposure to the hydrolysate (*p* = 0.034) as compared to medium alone (Figure 5b) was observed. Even though filaggrin and *SPRR* mRNA expression was consistently lower (Figure 5c), and *CCL26* and Calpain-14 mRNA expression was consistently greater in the IL-13-stimulated cells, it was not possible to identify consistent effects of the hydrolysate on these genes (data not shown). These results suggest that the hydrolysate may have a direct effect on aquaporin 3 and 11 gene expressions.

## 4. Discussion

Historically, the decreased exposure to intact protein antigens in milk through protein hydrolysation and the induction of oral tolerance are the two accepted mechanisms by which pHF-W reduces the risk of developing AD [3,33]. Prior studies suggest that reduced allergenicity may be a driver for the pHF-W effect in reducing the risk of AD [34] compared to IF. Nonetheless, the observation that some extensively hydrolysed formulas [35] or formula manufactured with another protein source such as soy [36] are not efficacious at reducing AD risk suggests that the avoidance of milk allergens is not sufficient to prevent AD. Secondly, it has been demonstrated that this pHF-W induces oral tolerance to the allergen BLG in milk sensitisation models [3,18], and prevents secondary allergic inflammation. However, intact protein (and thus the IF tested here) can induce oral tolerance to BLG using in vivo models; yet, in clinical settings, IF does not prevent the development of AD in infants [29,35,37]. The specific mode of action of pHF-W on skin barrier protection, in a model not dependent on a milk allergen, was thus the next logical hypothesis to test.

While the etiology of AD is still largely unknown, the evidence is mounting that a skin barrier defect precedes AD symptoms [5,9,10,12], and consecutively precedes food allergy development [38]. Both dry skin and increased TEWL in infants are associated with an increased risk of AD [14,39,40]. Since TEWL is a well-accepted indicator of skin barrier function in adults and babies and a marker for skin dehydration, it was selected as the most meaningful parameter for barrier status. The consistency of the data over multiple experiments confirmed the relevance of TEWL as the chosen marker. The effect of pHF-W or the hydrolysate on TEWL was not large but was consistently reproduced and significant.

To our knowledge, this is the first demonstration of a novel mechanism, unrelated to milk-specific oral tolerance induction, linking in vivo pHF-W to a skin benefit. Both pHF-W and its hydrolysate were able to reduce TEWL in this *Af* model. The beneficial modulation of the skin barrier seems to be attributable to the whey hydrolysate and potentially the peptides that compose it. It should be stressed that while our data suggest that this model is relevant for a reduction in risk markers such as TEWL, our model was not optimised for studying the prevention of allergic skin inflammation. Indeed, we were unable to develop a neonatal model in which skin symptoms of AD were prevented as a secondary effect following improved skin barrier function. Employing these models, AD symptoms were either too severe to be modulated in adult mice (data not shown) or TEWL dysregulation was insufficient to induce allergic skin inflammation in neonatal mice even with an additional *Af* skin exposure. The immature immune system in the neonatal mice [41] at the beginning of the experiment may explain why only low levels of *Af*-specific IgE were seen in our model. Altogether, our results with *Af* allergen confirm that Af has no predictive cross-reactivity with milk and that the benefit of the hydrolysed formula on the skin is distinct from an oral tolerance mechanism. Finally, the absence of association with a decrease in *Af*-specific sensitisation is in line with some of the human clinical data evidencing a reduction in AD risk but no change in milk sensitisation, allergic sensitisation, or food allergy prevalence [4].

Abnormal skin barrier function has been associated with the modulation of multiple genes [42]. Given the relationship between Th2 inflammation and skin barrier response, and the observation that IL-13 acts on keratinocytes in culture and skin to modify the expression of a number of these genes [43], we assessed the effect of formula on the expression of several barrier-related proteins in keratinocyte cultures in the presence and absence of IL-13 priming. Since filaggrin loss-of-function mutations were first described by Palmer et al. [5] as a major predisposing factor for AD, filaggrin has probably received the most attention for its contributions to skin barrier function, dysfunction, and TEWL [44,45,46,47,48,49,50,51]. As reported previously [43,52] and confirmed here, filaggrin expression is down-regulated in human IL-13 primed keratinocytes. However, in the present experiments, we were unable to discern the role for pHF-W or protein hydrolysate in modulating filaggrin expression. Additional analysis of filaggrin in the mouse experiments was not pursued as it is down-regulated in the atopic condition in humans but it is generally overexpressed in diseased mice [43,53]. This leads to difficulties in interpreting the murine data on filaggrin in the context of human skin diseases or skin barrier function.

Of the genes studied, aquaporins were likely candidates linked to TEWL as they facilitate the transfer of water. AQP3 is the main aquaporin in the skin, and functions as a water/glycerol channel [54]. AQP3 knockout mice exhibit reduced glycerol and water-holding content of the epidermis and delayed barrier recovery following the disruption of the stratum corneum [55]. In rats, the distribution of AQP3 expression has been associated with water loss changes occurring during skin maturation [32] and in two AD mouse models, increased AQP3 and TEWL were observed [55,56]. Furthermore, in both AD [57] and psoriasis [58], the disturbed regulation of AQP3 has been noted and is possibly related to skin dryness and increased TEWL in these diseases. The murine *Aqp3* mRNA expression was modulated in mice following hydrolysate supplementation. A reduction in human *AQP3* mRNA expression was also observed in vitro following pHF-W or hydrolysate stimulation. *AQP11* is essential for the proximal tubular function and has been described to maintain a slow but constant water movement across the membrane [59]. The skin and kidney are both involved in body water regulation. *AQP11 mRNA* expression was previously identified in primary keratinocytes [43]. Here, we show for the first time that *AQP11* is up-regulated (7-fold) by IL-13 and its expression is significantly decreased by the hydrolysate tested in vitro. The suppression of the increase in *AQP11* in IL-13 stimulated keratinocytes suggests that regulation of aquaporin channels may differently modulate water exchange during inflammation and upon hydrolysate supplementation. Of course, the in vitro results must be taken with caution considering that hydrolysate components were exposed directly onto primary human keratinocytes.

Most prevention studies with pHF-W have been performed in infants at increased allergy risk who had at least one first-degree relative with a history of allergy or atopic dermatitis [2]. Data generated in unselected, general-population infants show a similar effect size for decreased risk of developing AD following supplementation with pHF-W [29]. The allergic family history is thus not required for the effect, suggesting that nutritional intervention with pHF-W may counteract environmental factors. Interestingly, similar effects have been shown using skin ointment to hydrate and protect the skin from environmental exposure and dehydration [60]. As such, strategies aiming at reinforcing skin barrier function may be effective and our data suggest that nutritional intervention with pHF-W may be a viable alternative for strengthening the skin barrier by reducing TEWL. Further support for beneficial cutaneous effects by oral supplementation with hydrolysed protein can be found in the skincare field [61,62,63]. Several clinical trials have shown that oral supplementations with peptides from collagen were able to improve skin quality and hydration in adults based on biophysical measurement methods, including corneometry (skin conductance), elasticity, and TEWL, among others [61,62]. The in vivo improvement in the quality and quantity of collagen and improved skin hydration was underscored by a direct effect of collagen peptides on the glycosaminoglycan levels and collagen content in human skin explants [61]. Additionally, in a skin Ultraviolet B (UVB) irradiation model in vivo, whey peptides were shown to prevent type IV collagen degradation, angiogenesis, proliferation, and DNA damage [64]. Taken together, these results on the oral supplementation of hydrolysed collagen from multiple sources, including cattle, pigs, and fish, and our preclinical model results with partially hydrolysed whey protein suggest the possibility that specific or non-specific bioactive peptides provided orally can influence skin barrier and hydration properties. Yet, the mechanisms behind the benefit of oral peptides for the skin are largely unknown and the anti-inflammatory and anti-oxidative properties of whey peptides and their influence on inflammatory mediators [65], or their effect on the microbiota may also play a role in lessening barrier damage due to external stimuli.

## 5. Conclusions

In conclusion, the partially hydrolysed whey formula examined in this study has been shown to have reduced allergenicity [3], and to induce oral tolerance [3] in rodents. Here, we demonstrated a novel mechanism by which partially hydrolysed whey formula and its hydrolysate can reduce TEWL, modulate barrier-related gene expression notably AQP3 gene, and thus possibly improve skin barrier function in an *Af* model. We showed that the hydrolysate was responsible for the effect in vivo and that the direct regulation of gene expression by the whey hydrolysate on primary epithelial cells is possible. Further studies are warranted to better understand the cellular and molecular mechanisms involved in the protection of skin barrier dysfunction and AD development.

## Data Availability

All data and statistical reports can be obtained upon request.

## Abbreviations

Aquaporin-3 (AQP3), aquaporin-11 (AQP11), *Aspergillus fumigatus (Af)*, atopic dermatitis (AD), beta-lactoglobulin (BLG), bronchoalveolar lavage (BAL), cycle threshold (Ct), digoxigenin-3-O-methylcarbonyl-ε-aminocaproic acid-N-hydroxysuccinimide (DIG), glyceraldehyde 3-phosphate dehydrogenase (GAPDH), hypoxanthine phosphoribosyl-transferase (HPRT), interleukin-13 (IL-13), immunoglobulin (Ig), intact protein infant formula (IF), non-sensitised (N.S.), optical density (OD) partially hydrolysed whey-based infant formulas (pHF-W), sensitised (S.), small proline-rich protein (SPRR), T helper type 2 (Th2), tetramethylbenzidine (TMB), thymic stromal lymphopoietin (TSLP), transepidermal water loss (TEWL), ultraviolet B (UVB).
